# Alterations to oxidative stress markers in dogs after a short-term stress during transport*

**DOI:** 10.1017/jns.2014.47

**Published:** 2014-09-25

**Authors:** Chayanne S. Ferreira, Ricardo S. Vasconcellos, Raquel S. Pedreira, Flavio L. Silva, Fabiano C. Sá, Fernanda S. A. Kroll, Ana P. J. Maria, Katiani S. Venturini, Aulus C. Carciofi

**Affiliations:** 1 College of Agrarian and Veterinarian Sciences (FCAV), São Paulo State University (UNESP), Via de Acesso Professor Paulo Donato Castellane, s/n Jaboticabal, 14.884-900SP, Brazil; 2 Department of Animal Science (DZO), State University of Maringá (UEM), Av. Colombo, 5790, Maringá87020-900, PR, Brazil

**Keywords:** Alpha-tocopherol, Antioxidants, Canine nutrition, Lipoperoxidation, Thiobarbituric acid, αToc, α-tocopherol, DPPH, 2,2-diphenyl-1-picryl-hydrazyl, PC, protein carbonilation, Ret, retinol, ROS, reactive oxygen species, SH, total sulfhydryl groups, TAC, total antioxidant capacity, TBARS, thiobarbituric acid reactive substances

## Abstract

While methods to evaluate antioxidant capacity in animals exist, one problem with the models is induction of oxidative stress. It is necessary to promote a great enough challenge to induce measurable alterations to oxidative parameters while ensuring the protocol is compatible with animal welfare. The aim of the present study was to evaluate caged transport as a viable short-term stress that would significantly affect oxidative parameters. Twenty adult Beagle dogs, maintained on the same diet for 60 d prior to the transport, were included in the study. To simulate the stress, the dogs were housed in pairs in transport cages (1·0 m × 1·0 m × 1·5 m), placed on a truck coupled to a trailer and transported for a period of 15 min. Blood collection was performed immediately before and again 3 h after the transportation to evaluate oxidative parameters in blood serum, including thiobarbituric acid reactive substances (TBARS), total antioxidant capacity (TAC), sequestration activity of the radical 2,2-diphenyl-1-picryl-hydrazyl (DPPH•), protein carbonylation (PC), total sulfhydryl groups (SH), alpha-tocopherol (αToc) and retinol (Ret). PC, SH and αToc were not significantly changed in the study; however, TBARS, TAC and DPPH increased, whereas Ret decreased after the transport. Although the lack of a control group of dogs not submitted to transport is a limitation to be considered, we conclude that the transport model is effective in inducing an antioxidant response in dogs and relevant blood parameters show sensitivity to this proposed model.

Living with the risk of oxidative stress is the price that aerobic organisms must pay for their more efficient bioenergetics. Oxidative stress has generated scientific interest because of its generally accepted role as a major contributor to normal senescence as well as much other pathology with serious public health implications. The term ‘oxidative stress’ is used to define a group of interrelated phenomena that increase the generation of free radicals, especially reactive oxygen species (ROS) and the associated damage to cellular constituents^(^^1^^)^. Oxygen is essential for many living organisms for mitochondrial ATP production. It has been estimated that about 1–4% of the oxygen consumed by aerobes is converted to superoxide anion (O_2_^•−^) and hydrogen peroxide^(^^2^^)^. These two compounds are capable of generating even more reactive species such as hydroxyl radical (^•^OH), which can damage several intracellular macromolecules^(^^2^^)^.

To protect themselves against damage by free radicals, cells have a variety of antioxidant systems in different cellular compartments some of which are enzyme-based, while others are not^(^^3^^)^. All higher organisms have these complex antioxidant systems and their proper function is necessary for a healthy life. Catalase, glutathione peroxidase, superoxide dismutase, glutathione reductase and thioredoxin are the main antioxidant enzymes. Antioxidant enzymes can react directly or indirectly with ROS. Increases in antioxidant enzyme activities have been correlated to oxidative stress^(^^4^^–^^6^^)^. The non-enzymatic antioxidants act as free radical scavengers and include compounds such as vitamins C and E, glutathione, uric acid, albumin, bilirubin, *N*-acetylcysteine and melatonin^(^^7^^)^.

Stressful situations are associated with increased cortisol and catecholamine production and oxidative stress. Some stressors that have been studied in dogs are noise, training, immobilisation, novelty, transport and restricted housing conditions^(^^8^^)^. Oxidative stress may result in an imbalance between free radical production and the existing antioxidant capacity of the body. A decrease in antioxidant capacity may be associated with increased ROS values, while a decrease in ROS formation is often due to an increase in antioxidant capacity. This balance, however, has not always been shown^(^^9^^,^^10^^)^. In order to better explain the antioxidant response to stress, and the effect of dietary interventions to mitigate it, it is important to identify a study model that allows for understanding the relationships between stress and oxidative markers in the serum^(^^11^^)^. Such a model must allow for the induction of measurable alterations to oxidative parameters while remaining compatible with animal welfare standards^(^^8^^)^.

Considering this, the aim of the present study was to evaluate alterations to specific oxidative stress parameters in dogs after induction of short-term stress by transporting the animals in cages. We expect that stress induction will result in reduction of endogenous antioxidants and induction of the enzymatic antioxidant defence system, suggesting the organism is modifying antioxidant capacity in order to prevent increased concentrations of circulating oxidative metabolites in the blood.

## Experimental methods

All experimental procedures were approved by the Ethics Committee on Animal Use of the College of Agrarian and Veterinarian Sciences, São Paulo State University (Protocol: 010074/11).

### Animals, diets and study design

Twenty adult Beagles on average 4·9 years old (sem 2·8) and weighing on average 12·4 kg (sem 1·5) were used. The initial body condition score of the dogs was a 5 on a 9-point scale^(^^12^^)^. The health of all animals was confirmed prior to the start of the study through physical examinations and laboratory analyses (complete blood count, urea, creatinine, alkaline phosphatase, aspartate aminotransferase and albumin).

During the experiment the dogs were housed in 1·5 m × 4·5 m kennels with a solarium. Drinking water was available *ad libitum*. The dogs were allowed to exercise and socialise daily with other dogs and people for at least 2 h in a grass playground (200 m^2^). The animals received the same extruded kibble diet during the 60 d of the experiment. This period was used to standardise the oxidative status of the dogs. The extruded diet was balanced for dog maintenance^(^^13^^)^, and contained the following chemical composition (per 4·184 kJ of metabolisable energy): crude protein, 66·7 g; acid-hydrolysed fat, 37·0 g; crude fibre, 4·8 g; ash, 16·1 g; nitrogen-free extract, 113·4 g; vitamin E, 50 mg; selenium, 0·075 mg. The diet included the following ingredients (per kg, as-fed basis): maize, 494·4 g; poultry by-product meal, 261·1 g; maize gluten meal, 50 g; wheat bran, 50 g; poultry fat, 95·2 g; choline chloride, 2·0 g; potassium chloride, 4·4 g; salt, 5·0 g; mineral and vitamin premix, 2·0 g; sunflower oil, 5·0 g; fish oil, 20·5 g; palatant enhancer, 10·0 g; antioxidant butylated hydroxytoluene, 0·2 g; calcium propionate, 1·0 g. The dogs were fed to maintain constant body weight during the experiment. To this end the dogs were fed individually calculated amounts once a day at 10:30 h and allowed them to eat for 30 min. After this time any remaining food was removed, weighed and the intake was recorded. The amount of provided food was initially calculated from the food's metabolisable energy content, which was estimated from its chemical composition, and the dogs were fed their recommended energy requirements for maintenance^(^^14^^)^. After the first week the dogs were weighed weekly and their food was adjusted to achieve constant body weight.

At the end of the diet adaptation period (60th d), the animals were transported for a short time to induce physiological stress. The dogs were housed in pairs in 1·0 m × 1·0 m × 1·5 m transport cages, placed on a truck coupled to a trailer and transported for 15 min. The transport started at 14:00 h, and the average day temperature was 31·3°C and the relative air humidity was 45%. The animals had not been previously exposed to this procedure. To evaluate oxidative parameters, blood was collected from the jugular vein by direct puncture at two times: immediately before and 3 h after the transportation. The blood was placed in vacuum glass tubes without anticoagulant and maintained in the dark, under ice. Immediately after collection, blood was centrifuged at 500 ***g*** and 4°C for 10 min to extract the serum and then frozen at −80°C for future analysis.

### Laboratory analysis

The serum samples were used to measure thiobarbituric acid reactive substances (TBARS), total antioxidant capacity (TAC), 2,2-diphenyl-1-picryl-hydrazyl (DPPH) sequestration capacity by the antioxidants present in the sample, protein carbonylation (PC), total sulfhydryl groups (SH), and α-tocopherol (αToc) and retinol (Ret) contents.

TBARS concentrations were determined as described by Payá *et al.*^(^^15^^)^ with modifications. A serum sample (200 µl) was added to 2 ml TBARS solution (15 % TCA, 0·275 % thiobarbituric acid and 0·25 m hydrochloric acid). Then the tube was boiled for 15 min in a water bath, cooled for 15 min on ice and centrifuged for 15 min at 1200 ***g*** and 4°C. The supernatant was analysed by a UV–visible spectrophotometer. Chromogen was identified by reading absorbance at a fixed wavelength of 532 nm (Labquest, Labtest Diagnostica). The results were expressed in absorbance.

The analysis of TAC was performed by use of a commercial kit (Antioxidant Assay Kit, CS0790, Sigma-Aldrich). The principle of this procedure is to induce *in vitro* formation of the free radicals ferril myoglobin, metmyoglobin and hydrogen peroxide to oxidise 2,2-azino-bis(3- ethylbenzthiazoline-6-sulfonic acid leading to the production of the radical cation 2,2-azino-bis(3- ethylbenzthiazoline-6-sulfonic acid^•+^, a soluble, greenish chromogen that can be determined by the spectrophotometer at a wavelength of 405 nm. Antioxidants present in the sample partially inhibit 2,2-azino-bis(3- ethylbenzthiazoline-6-sulfonic acid oxidation, and the results were expressed in Trolox equivalents.

The hydrogen donor activity by serum antioxidants was evaluated by DPPH• quenching read by UV–visible spectrophotometry^(^^16^^)^. A mixture of 0·5 ml serum and 0·5 ml acetone were vortexed for 1 min and then centrifuged for 5 min at 5500 ***g*** and 4°C for deproteinisation of the sample. The supernatant was filtered with a Pasteur pipette filled with cotton cloth to remove small particles. A 0·1 mm methanolic DPPH solution (0·0039 g per 100 ml) was prepared immediately before testing and was incubated in the dark. An aliquot of 400 µl of DPPH solution was added to 360 µl of phosphate buffer (pH 7·4) and 40 µl of sample and homogenised by vortexing. Absorbance was read at 505 nm (Labquest, Labtest Diagnostica) at 0, 5, 10, 15 and 20 min after mixing. The inhibition (discoloration) of DPPH• radical was calculated as the relative percentage of absorbance of the sample at the time of the reading compared with a blank (400 µl of DPPH solution plus 400 µl of phosphate buffer).

Protein carbonylation was assayed using a commercial kit (Protein Assay Kit Colorimetric Carbonyl, Cayman Chemical Company), which uses the reaction of 2,4-dinitrophenylhydrazine with the carbonyl groups in proteins to produce a compound that can be quantified by colorimetry. Serum samples were used according to the manufacturer's description.

The measurement of total thiol, or sulfhydryl groups (SH), was performed according to the method described by Hu^(^^17^^)^. By this method the thiol groups react with 5,5′-dithiobis-2-nitrobenzoic acid forming a highly coloured anion with a maximum absorption at 412 nm. The SH concentration was calculated using reduced glutathione as a standard and the results were expressed in mm/l.

Analyses of αToc and Ret were performed according to the methodology described by Arnaud *et al.*^(^^18^^)^ Serum samples (200 µl) were added to 400 µl of an ethanol and hexane mixture (1 : 1). After vortexing for 1 min, the sample was centrifuged at 4000 ***g*** for 10 min and dried in a stream of N_2_ gas. The sample was suspended in 200 µl of the mobile phase solution and analysed by HPLC (Shimadzu model LC- 20AT; column type C – 18150×4·6 mm–5 µm; UV–visible detector SPD- 20A model). The mobile phase consisted of acetonitrile:dichloromethane:methanol at a ratio of 7:2:1, the flow rate was 1·0 ml/min and detection was performed at 292 and 352 nm for αToc and Ret, respectively. The concentrations were determined by the use of external standards and results were expressed as μmol/l of serum.

### Statistical analysis

The data were analysed in a completely randomised design using the general linear model procedures of the Statistical Analysis Systems statistical software package version 9.0 (SAS Institute, Cary, NC, USA). The experimental unit was one dog. Data were first analysed for normal distribution and variance equality. Repeated measures ANOVA was performed to determine the effect of transport and values were considered significant at *P* < 0·05.

## Results

The short period of transport was effective in inducing measurable physiological stress in the dogs. TBARS (*P* = 0·046), TAC (*P* = 0·007) and DPPH (*P* = 0·006) increased, while Ret decreased (*P* = 0·024) after the transport. There was not a significant change (*P* > 0·05) in the concentrations of PC, SH or αToc (Table 1).

**Table 1.** Serum oxidative parameters in dogs before and after physiological stress induction by short-term transport

	Serum concentration			
Parameter	Before	After 3 h	sem*	*P*
TBARS (absorbance)	0·040	0·048	0·003	0·043
DPPH (% decolouration)	5·070	6·195	0·311	0·006
TAC (EqTROLOX)	0·422	0·552	0·039	0·007
PC (nmol/mg)	17·03	20·63	2·40	0·280
SH (μmol/l)	48·60	51·44	1·56	0·150
αToc (μmol/l)	52·13	48·37	3·23	0·353
Ret (μmol/l)	2·30	1·86	0·153	0·024

TBARS, thiobarbituric acid reactive substances; TAC, total antioxidant capacity; DPPH,2,2-diphenyl-1-picryl-hydrazyl sequestration capacity by the antioxidants present in the sample; PC, protein carbonilation; SH, total sulfhydryl groups; αToc, α-tocopherol; Ret, retinol.

*Standard error of the mean, *n* 20 dogs.

## Discussion

Oxidative stress reflects a cellular imbalance where ROS production exceeds the antioxidant capacity to neutralise the ROS and can result in oxidative damage to nearby molecules such as DNA, RNA and lipids^(^^11^^)^. In the present study, short-term stress appeared to activate antioxidant defence mechanisms in dogs and thus it was possible to verify the involvement of some serum markers in an antioxidant response (increase of the oxidative metabolites and antioxidant capacity and decrease of the endogenous antioxidants).

Studies have shown that cortisol levels are closely related to lipid, RNA and DNA oxidation during short-term stress. The positive association between serum TBARS and cortisol levels, for example, has already been shown for calves after transport by truck^(^^19^^)^. The serum cortisol peak occurs approximately 15–30 min after stress induction^(^^11^^)^. Psychological factors directly affect the hypothalamic–pituitary–adrenocortical axis, which regulates cortisol's release. However, the hypothalamic–pituitary–adrenocortical axis is affected differently according to the stressor involved. When stress is detected this information is sent to the thalamus and prefrontal cortex, which are the brain centres that analyse the significance of this environmental stimulation to the body. The cognitive response by the central nervous system can generate an emotional response via complex communications between the prefrontal cortex and limbic system, which will activate the hypothalamic–pituitary–adrenocortical axis^(^^20^^)^. In dogs several stressors have been identified^(^^21^^)^. In the present study, the cages, transport vehicle and noise served as the stressors. Although we did not quantify serum cortisol in the dogs as a marker of stress, a limitation that need be considered, the protocol seems to have been effective for stress induction given the responses of most of the oxidative parameters we analysed.

The conversion of oxygen during normal metabolism to ROS may occur due to successive additions of electrons to the molecule during ATP production. This can occur when the demand for oxygen by the body is increased without prior adaptation such as in exhaustive exercise^(^^22^^)^ or short-term stress^(^^23^^)^. The ROS production can induce the formation of lipid peroxidation products^(^^24^^)^ and oxidised proteins^(^^23^^)^. Although the use of blood metabolites in the present study precludes the identification of specific tissue effects, we observed increased lipid and protein peroxidation (TBARS and PC, respectively), increased antioxidant capacity (TAC and DPPH) and a reduction in the concentrations of compounds with antioxidant activity (Ret and αToc, although the reduction in αToc was NS).

The increases in the antioxidant capacity indicators (TAC and DPPH) after the transport in the present study may have occurred due to the activation of antioxidant defence systems. Approximately 47·3% of the antioxidant capacity quantified by TAC depends on the concentrations of albumin and uric acid^(^^25^^)^, whereas the remainder depends on other molecules such as enzymes and low-molecular-weight antioxidants. Enzymatic compounds (catalase, superoxide dismutase, glutathione peroxidase), macromolecules (albumin, ceruloplasmin and ferritin) and low-molecular-weight substances (αToc, ascorbic acid, ubiquinol-10, β-carotene, uric acid, methionine, reduced glutathione and bilirubin) are mainly responsible for the serum TAC activity^(^^25^^)^. Since we determined the individual levels of only some of those compounds, it is difficult to know precisely which are responsible for the TAC elevation we observed. In any case, as expected serum antioxidant compounds were consumed while oxidation products and TAC increased simultaneously. The increase in TAC may be due to the activation of enzymatic systems or macromolecule production. The macromolecules with high antioxidant activity are the acute phase proteins produced and released by the liver, but these molecules usually increase in serum 4–5 h after stimulus^(^^26^^)^, and our blood samples was collected 3 h after stress stimulation. Given this, we believe activation of enzymatic antioxidant systems probably accounts for the TAC increase in the present study.

The reduction in Ret after stress was expected. We also observed the concentration of αToc was reduced, although not significantly. An inverse relationship between plasma concentrations of αToc and Ret with markers of lipid peroxidation has been demonstrated^(^^27^^)^, indicating these antioxidants are consumed during lipid peroxidation. Retinoids participate intracellularly in two steps of enzymatic oxidative reactions in which Ret is first converted to retinaldehyde and then to retinoic acid, and it is known that vitamin A liver reserves can be depleted after stress^(^^28^^)^. The reduced Ret concentrations in our dogs after the stressor were likely due to these reactions.

In the present study, we show that it is possible to measure and track changes in serum markers of oxidative status in Beagle dogs after induction of short-term transport stress. Our hypothesis was confirmed and the model was effective in inducing oxidative modification to several parameters. Lipid peroxidation increased and the consumption of the antioxidant Ret occurred during this period. Despite this, the enzymatic and cellular antioxidant defence systems responded effectively as measured through increases to antioxidant capacity (DPPH and TAC). However, one limitation to be considered is the lack of a control group of dogs that were not submitted to transport. Thus it is not possible to differentiate between the influences of normal daily variations that may occur due to circadian rhythms and the induced stress on serum antioxidants findings. Nevertheless, the proposed method proved to be convenient and capable of detecting alterations in oxidative status. This model can be used to stimulate the antioxidant system and may be of particular use in nutritional studies on dietary compounds with antioxidant properties, in which the antioxidant systems need to be challenged in order for the efficacy of the antioxidant supplements to be adequately evaluated.
